# Mitochondrial and nuclear ribosomal DNA dataset supports that *Paramphistomum leydeni* (Trematoda: Digenea) is a distinct rumen fluke species

**DOI:** 10.1186/s13071-015-0823-4

**Published:** 2015-04-02

**Authors:** Jun Ma, Jun-Jun He, Guo-Hua Liu, Dong-Hui Zhou, Jian-Zhi Liu, Yi Liu, Xing-Quan Zhu

**Affiliations:** State Key Laboratory of Veterinary Etiological Biology, Key Laboratory of Veterinary Parasitology of Gansu Province, Lanzhou Veterinary Research Institute, Chinese Academy of Agricultural Sciences, Lanzhou, Gansu Province 730046 PR China; College of Veterinary Medicine, Hunan Agricultural University, Changsha, Hunan Province 410128 PR China; Institute of Livestock Research, Tibet Academy of Agricultural and Animal Husbandry Sciences, Lhasa, Tibet Autonomous Region 850009 PR China; Jiangsu Co-innovation Center for the Prevention and Control of Important Animal Infectious Diseases and Zoonoses, Yangzhou University College of Veterinary Medicine, Yangzhou, Jiangsu Province 225009 PR China

**Keywords:** *Paramphistomum leydeni*, *Paramphistomum cervi*, Mitochondrial genome, Nuclear ribosomal DNA, Phylogenetic analysis

## Abstract

**Background:**

Rumen flukes parasitize the rumen and reticulum of ruminants, causing paramphistomiasis. Over the years, there has been considerable debate as to whether *Paramphistomum leydeni* and *Paramphistomum cervi* are the same or distant species.

**Methods:**

In the present study, the complete mitochondrial (mt) genome of *P. leydeni* was amplified using PCR-based sequencing and compared with that of *P. cervi*. The second internal transcribed spacer (ITS-2) of nuclear ribosomal DNA (rDNA) of *P. leydeni* specimens (n = 6) and *P. cervi* specimens (n = 8) was amplified and then sequenced. Phylogenetic relationship of the concatenated amino acid sequence data for 12 protein-coding genes of the two rumen flukes and selected members of Trematoda was evaluated using Bayesian inference (BI).

**Results:**

The complete mt genome of *P. leydeni* was 14,050 bp in size. Significant nucleotide difference between the *P. leydeni* mt genome and that of *P. cervi* (14.7%) was observed. For genetic divergence in ITS-2, sequence difference between *P. leydeni* and *P. cervi* was 3.1%, while no sequence variation was detected within each of them. Phylogenetic analysis indicated that *P. leydeni* and *P. cervi* are closely-related but distinct rumen flukes.

**Conclusions:**

Results of the present study support the proposal that *P. leydeni* and *P. cervi* represent two distinct valid species. The mt genome sequences of *P. leydeni* provide plentiful resources of mitochondrial markers, which can be combined with nuclear markers, for further comparative studies of the biology of *P. leydeni* and its congeners from China and other countries.

**Electronic supplementary material:**

The online version of this article (doi:10.1186/s13071-015-0823-4) contains supplementary material, which is available to authorized users.

## Background

Species of *Paramphistomum* (Trematoda: Digenea), known as the ‘rumen flukes’ or ‘amphistomes’, are the pathogens of paramphistomiasis of ruminants, such as cattle, buffalo, sheep, goat and deer [1-5]. Although rumen flukes are considered neglected parasites, they are widely distributed in many continents of the world, (e.g., Asia, the Americas, Europe, Africa and Oceania) [1,2,4,6-12]. Rumen flukes require aquatic snails as intermediate hosts and the pre-parasitic stages of miracidia and stages in snails (sporocyst, redia and cercaria) are similar to those of liver flukes, such as *Fasciola hepatica* [13]. Cercaria escape from snails and attach to aquatic plants forming infectious metacercaria. Ruminants acquire infection through ingestion of infectious metacercaria attached to plants. Infection with adult *Paramphistomum* can cause chronic clinical signs, such as emaciation, anemia, diarrhea and edema [8]. The immature paramphistomes might migrate through intestine towards rumen, reticulum, abomasums, bile duct and gallbladder. The migration could lead to significant morbidity in ruminants, even death.

*Paramphistomum leydeni* and *Paramphistomum cervi* are common rumen flukes in many countries [1,2], particularly in Argentina [3]. Various host animals are often infected concurrently with *P. leydeni*, *P. cervi* and other paramphistomums globally, and the host or geographical preference of the two rumen flukes has not been documented. In spite of the economic loss and morbidity of paramphistomiasis, over the years, there has been a significant controversy as to whether *P. leydeni* and *P. cervi* represent the same or distinct fluke species. The taxonomy of *P. leydeni* and *P. cervi* is still unclear [1]. Although the amphistome species are morphologically very similar [2], reports have documented that *P. leydeni* and *P. cervi* are morphologically distinct species based on morphological features of the adult (e.g., genital opening type, pharynx type, ventral pouch and tegumental papillae absent or present) [13,14]. Furthermore, some studies have shown that *Cotylophoron cotylophorum* was re-classified as *P. leydeni* [1,2,5]. *P. leydeni*, as well as *Paramphistomum hiberniae*, *Paramphistomum scotiae* and *Cotylophoron skriabini*, was regarded as established synonym of *P. cervi* [5,14-17].

Molecular tools, using genetic markers in mitochondrial (mt) DNA and in the internal transcribed spacer (ITS) regions of nuclear ribosomal DNA (rDNA), have been used effectively to identify trematode species [18-21]. For rumen flukes, Yan et al. (2013) reported that mtDNA might be an useful molecular marker for studies of inter- and intra-specific differentiation of the Paramphistomidae [21]. Additionally, the ITS-2 rDNA has also proved to be a valuable marker for identification of amphistomes [1,2]. Advancements in long PCR-coupled sequencing and bioinformatic methods are providing effective approaches to probe into the biology of these parasites [22,23]. Therefore, in the present study, the complete mt genome of *P. leydeni,* and ITS-2 rDNA sequences of *P. leydeni* and *P. cervi* were sequenced, analyzed and compared to test the hypothesis that *P. leydeni* and *P. cervi* are two genetically distinct species.

## Methods

### Ethics statement

This study was approved by the Animal Ethics Committee of Lanzhou Veterinary Research Institute, Chinese Academy of Agricultural Sciences. Adult specimens of *Paramphistomum* were collected from bovids and caprids, in accordance with the Animal Ethics Procedures and Guidelines of the People’s Republic of China.

### Parasites, total genomic DNA extraction and the ascertainment of specimen identity

Adult specimens of *Paramphistomum* were collected, *post-mortem*, from the rumens of naturally infected goats in Nimu County, Tibet Autonomous Region; from livers and rumens of naturally infected yaks in Tianzhu and Maqu counties, Gansu Province; Ruoergai County, Sichuan Province; and Shaoyang City, Hunan Province, China. Samples were washed in physiological saline extensively, fixed in 70% (v/v) ethanol and preserved at −20°C until use.

Because the specimens were kept in 70% ethyl alcohol, it was difficult to acquire the accurate morphological data of the paramphistomums, thus molecular identification was performed to ascertain the identities of the two paramphistomums. Total genomic DNA of each sample was extracted separately by sodium dodecyl sulfate (SDS)/proteinase K digestion system [24] and mini-column purification (Wizard-SV Genomic DNA Purification System, Promega) according to the existing instructions.

ITS-2 rDNA of individual *Paramphistomum* specimens was amplified by PCR and sequenced according to established methods [25-27], and the identity of individual *Paramphistomum* specimens was ascertained by comparison with corresponding sequences available in GenBank [2].

### Long-range PCR-based sequencing of mt genome

The primers (Table 1) were designed to relatively conserved regions of mtDNA nucleotide sequences from *P. cervi* and other closely-related taxa. The mt DNA was amplified from one specimen of *P. leydeni* collected from a goat in Nimu County, Tibet Autonomous Region, China. The full mt genome of *P. leydeni* was amplified in 4 overlapping long fragments between *cox*3 and *atp*6 (approximately 3.5 kb), between *atp*6 to *cox*1 (approximately 4 kb), between *cox*1 to *rrn*S (approximately 2.6 kb) and between *rrn*L to *cox*3 (approximately 5.5 kb) (Table 1). PCR reactions were conducted in a total volume of 50 μl using 4 mM MgCl_2_, 0.4 mM each of dNTPs, 5 μl 10× LATaq buffer, 5 mM of each primer, 0.5 μl LA Taq DNA polymerase (Takara, Dalian, China) and 2 μl DNA templates in a thermocycler (Biometra, Göttingen, Germany). The PCR cycling conditions began with an initial denaturation at 92°C for 2 min, then 12 cycles of denaturation at 92°C for 20 s, annealing at 55–62°C for 30 s and extension at 60°C for 3–5 min, followed by 92°C denaturation for 2 min, plus 28 cycles of 92°C for 20 s (denaturation), 55–62°C for 30 s (annealing) and 66°C for 3–5 min, with 10 min of the final extension at 66°C. A cycle elongation of 10 s was added for each cycle. A negative control containing nuclease-free water was included in every amplification run. Each amplicon (4 μl) was evidenced by electrophoresis in a 1.2% agarose gel, stained with Gold View I (Solarbio, Beijing, China) and photographed by GelDoc-It TS^TM^ Imaging System (UVP, USA). Amplified products were sent to Genewiz Company (Beijing, China) for sequencing using ABI3730 sequencer from both directions using the primer walking strategy [28]. Sequencing results were tested by Seq Scanner 2 and artificial secondary interpretation was performed by professional technical personnel to ensure that the fragment of 50–800 bp of each sequencing result was read accurately. The walking primers were designed for approximately 600 to 700 bp of each sequence to assure the accuracy of two adjacent sequencing reactions by the sequencing company. The sequences were assembled manually to avoid errors by visualization of the chromatograms.

**Table 1:** **Sequences of primers used to amplify long PCR fragments of** ***Paramphistomum leydeni***

**Primer**	**Sequence (5’-3’)**	**Size (kb)**	**Amplified region**
Pl1F	GCGGTATTGGCATTTTGTTGATTA	~3.5	Partial *cox*3-H-*cyt*b-SNCR-*nad*4L-*nad*4
Pl1R	CATCAAGACAACAGGACGCACTAAAT		-Q -F-M-partial *atp*6
Pl2F	GGAAGTTAGGTGTTTGGAATGTTG	~4.0	Partial *atp*6-*nad*2-V-A-D-*nad*1-N-P-I-K
Pl2R	CCAAACAATGAATCCTGATTTCTC		-*nad*3-S1-W-partial *cox*1
Pl3F	TTTTTTGGGCATAATGAGGTTTAT	~2.6	Partial *cox*1-T-*rrn*L-C-partial *rrn*S
Pl3R	CCAACATTACCATGTTACGACTT		
Pl4F	GGAGCAAGATACCTCGGGGATAA	~5.5	Partial *rrn*L-C-r*rn*S-*cox*2-*nad*6-Y-L1-S2-L2
Pl4R	CCCACCTGGCTTACACTGGTCTTA		-R-*nad*5-G-E-LNCR-*cox*3-H-partial *cyt*b

### Amplification and sequencing of ITS-2 rDNA

The ITS rDNA region, spanning partial 18S, complete ITS-1, complete 5.8S, complete ITS-2 and partial 28S rDNA sequences, was amplified from the extracted DNA of each specimens using primers 18SF (forward; 5’-CACCGCCCGTCGCTACTACC-3’) and 28SR (reverse; 5’-ACTTTTCAACTTTCCCTC-3’) described previously [27]. The amplicons were approximately 2582 bp in length.

### Assembling, annotation and bioinformatic analysis

*P. leydeni* mtDNA sequences were assembled manually and aligned against the whole mt DNA sequences of *P. cervi* (KF_475773) [21] and *Paragonimus westermani* (AF_219379) using MAFFT 7.122 to define specific gene boundaries. Twelve protein-coding genes were translated into amino acid sequences using MEGA 6.06 selecting the trematode mt genetic code option. The tRNA genes were identified using the program tRNAscan-SE [29] and ARWEN (http://130.235.46.10/ARWEN/) or by visual inspection [30]. The two rRNA genes were annotated by comparison with those of *P. cervi* and *P. westermani*.

### Sliding window analysis of nucleotide variability

Pairwise alignment of the complete mt genomes of *P. leydeni* and *P. cervi*, including tRNAs and all intergenic spacers, was conducted by MAFFT 7.122 to locate variable nucleotide sites between the two rumen flukes. A sliding window analysis (window length =300 bp, overlapping step size =10 bp) was performed using DnaSP v. 5 [31] to estimate nucleotide diversity Pi (π) for each mt genes in the alignment. Nucleotide diversity was plotted against mid-point positions of each window, and gene boundaries were identified.

### Phylogenetic analysis

For comparative purposes, the concatenated amino acid sequences conceptually translated from individual genes of the mt genomes of the two rumen fluke were aligned with published mt genomes from selected Digenea, including *Clonorchis sinensi*s (FJ_381664) [32], *Opisthorchis felineus* (EU_921260) [32] and *Opisthorchis viverrini* (JF_739555) [33] [family Opisthorchiidae]; *Haplorchis taichui* (KF_214770) [34] [Heterophyidae]; *P. westermani* (AF_219379) [Paragonimidae]; *Fasciola hepatica* (NC_002546) [35], *Fasciola gigantica* (NC_024025) [19] and *Fasciola* sp. (KF_543343) [19] [Fasciolidae]; *Dicrocoelium chinensis* (NC_025279) [20] and *Dicrocoelium dendriticum* (NC_025280) [20] [Dicrocoeliidae] and *P. cervi* (KF_475773) [21] [Paramphistomidae]. The sequence of *Schistosoma turkestanicum* (HQ_283100) [36] [Schistosomatidae] was included as an outgroup.

All amino acid sequences were aligned using MAFFT 7.122 and excluding ambiguously aligned regions using Gblocks v. 0.91b selecting the defaults choosing options for less strict flanking positions. Then the alignment was modified into nex format and subjected to phylogenetic analysis using Bayesian inference (BI) applying the General Time Reversible (GTR) model as described previously [37]. Four Monte Carlo Markov Chain (MCMC) were run and two independent runs for 10000 metropolis-coupled MCMC generations were used, sampling a tree every 10 generation in MrBayes 3.1.2. Phylograms were viewed using FigTree v. 1.42 [38].

## Results and discussion

### Identity of *P. leydeni* and *P. cervi*

The ITS-2 sequences of *P. leydeni* specimens (n = 6) (GenBank accession nos. KP341666 to KP341671) were 100% homologous to previously published sequences of *P. leydeni* from sheep and cattle in Buenos Aires and Entre Ríos provinces, Argentina (HM_209064 and HM_209067), deer in Ireland (AB_973398) and ruminants in northern Uruguay (KJ_995524 to KJ_995529). The ITS-2 sequences of *P. cervi* specimens (n = 8) (GenBank accession nos. KP341658 to KP341665) were 100% identical to those of *P. cervi* from cattle in Heilongjiang Province, China (KJ_459934, KJ_459935).

### Content and organization of mt genome of *P. leydeni*

**Figure 1:**
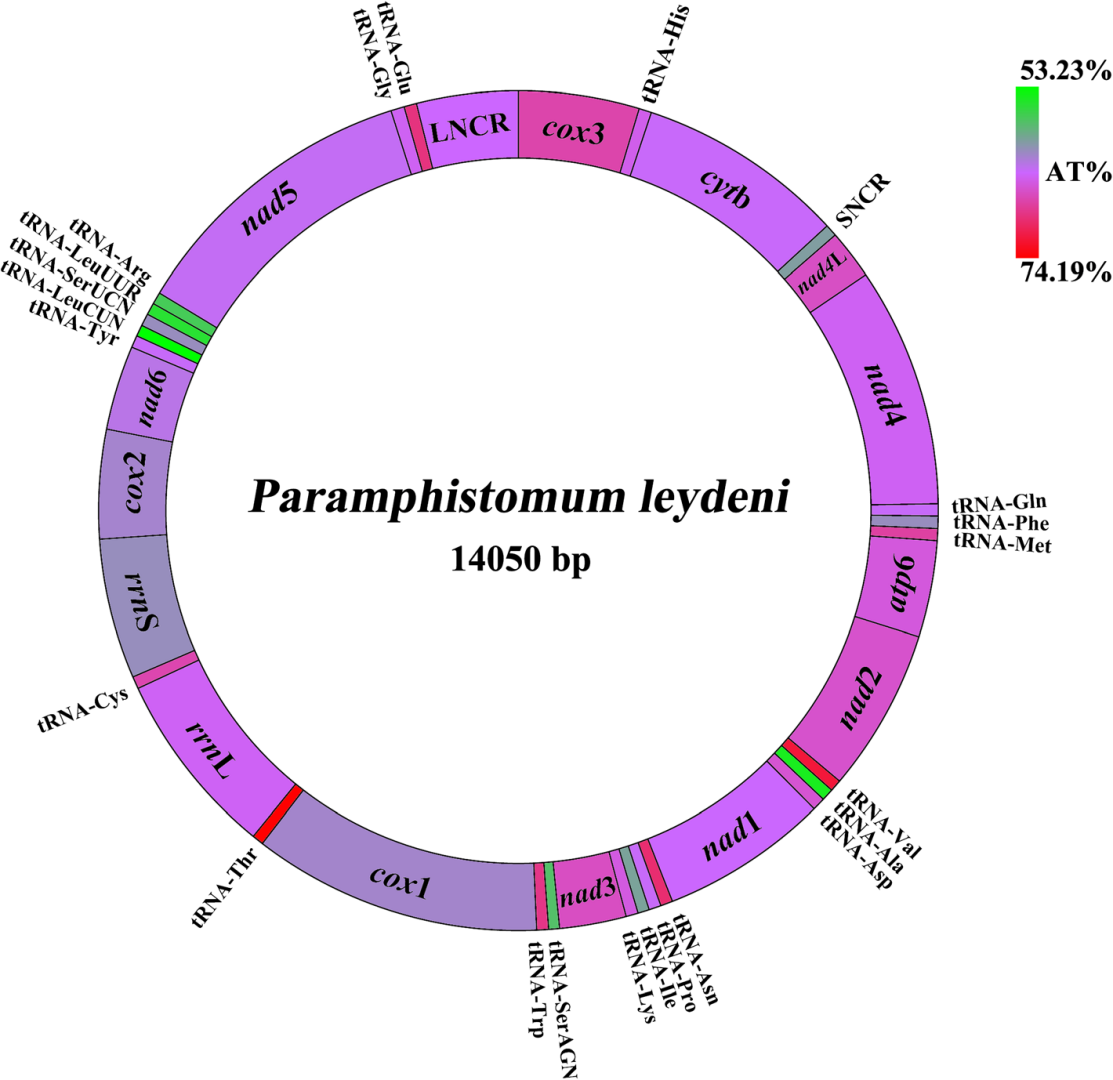
**Organization of the mitochondrial genome of** ***Paramphistomum leydeni.*** The scale is accurate. All genes are transcribed in the clockwise direction, and use standard nomenclature including 22 tRNA genes. “LNCR” and “SNCR” refer to a large non-coding region and small non-coding region. The A + T content also showed in each gene or region and represented by color.

The complete mt genome sequence of *P. leydeni* (GenBank accession no. KP341657) is 14,050 bp in size, 38 bp larger than that of *P. cervi*. The circular genome of *P. leydeni* contains 36 genes that transcribing in the same direction, covering 12 protein-coding genes (*nad*1–6, *nad*4L, *cox*1–3, *cyt*b and *atp*6), 22 tRNA genes and two rRNA genes (*rrn*L and *rrn*S) (Table 2) which is consistent with those of all the trematode species available to date (Figure 1) [18-21,32,33,36,39,40]. A comparison of nucleotide sequences of each protein coding gene, the amino acid sequences, two ribosomal DNA genes and two NCRs is given in Tables 2 and 3.

**Table 2:** **The features of the mitochondrial genomes of** ***Paramphistomum leydeni*** **(PL) and** ***Paramphistomum cervi*** **(PC)**

**Gene**	**Positions and nt sequence sizes (bp)**		**Start and stop codons**		**tRNA Anti-codons**		**Intergenic nt (bp)**	
	**PL (5’-3’)**	**PC (5’-3’)**	**PL**	**PC**	**PL**	**PC**	**PL**	**PC**
*cox*3	1-645 (645)	1-645 (645)	ATG/TAG	ATG/TAG			0	0
tRNA-His (H)	647-714 (68)	647-715 (69)			GTG	GTG	1	3
*cyt*b	717-1829 (1113)	720-1832 (1113)	ATG/TAG	ATG/TAG			2	4
SNCR	1830-1894 (64)	1833-1890 (58)					0	0
*nad*4L	1895-2158 (264)	1891-2154 (264)	ATG/TAG	ATG/TAG			0	0
*nad*4	2119-3399 (1281)	2115-3395 (1281)	GTG/TAG	GTG/TAG			−40	−40
tRNA-Gln (Q)	3404-3469 (66)	3398-3462 (65)			TTG	TTG	4	2
tRNA-Phe (F)	3501-3567 (67)	3489-3553 (65)			GAA	GAA	31	26
tRNA-Met (M)	3565-3629 (65)	3553-3615 (63)			CAT	CAT	−3	−1
*atp*6	3630-4145 (516)	3616-4131 (516)	ATG/TAG	ATG/TAG			0	0
*nad*2	4153-5025 (873)	4139-5011 (870)	ATA/TAG	GTG/TAG			7	7
tRNA-Val (V)	5049-5112 (64)	5014-5077 (64)			TAC	TAC	23	2
tRNA-Ala (A)	5122-5187 (66)	5085-5154 (70)			TGC	TGC	9	7
tRNA-Asp (D)	5197-5266 (70)	5165-5229 (65)			GTC	GTC	9	10
*nad*1	5269-6165 (897)	5233-6129 (897)	ATG/TAG	ATG/TAG			2	3
tRNA-Asn (N)	6170-6235 (66)	6142-6207 (66)			GTT	GTT	4	12
tRNA-Pro (P)	6235-6300 (66)	6208-6270 (63)			TGG	TGG	−1	0
tRNA-Ile (I)	6302-6363 (62)	6272-6334 (63)			GAT	GAT	1	1
tRNA-Lys (K)	6370-6435 (66)	6344-6409 (66)			CTT	CTT	6	9
*nad*3	6436-6792 (357)	6410-6766 (357)	ATG/TAG	ATG/TAG			0	0
tRNA-Ser (S1)	6810-6868 (59)	6785-6843 (59)			GCT	GCT	17	18
tRNA-Trp (W)	6878-6941 (64)	6853-6915 (63)			TCA	TCA	9	9
*cox*1	6942-8486 (1545)	6916-8460 (1545)	ATA/TAG	GTG/TAG			0	0
tRNA-Thr (T)	8500-8561 (62)	8470-8534 (65)			TGT	TGT	13	9
*rrn*L	8562-9556 (995)	8535-9520 (986)					0	0
tRNA-Cys (C)	9557-9623 (67)	9527-9586 (60)			GCA	GCA	0	6
*rrn*S	9624-10372 (749)	9592-10340 (749)					0	5
*cox*2	10373-10954 (582)	10341-10919 (579)	ATG/TAG	ATG/TAG			0	0
*nad*6	10948-11400 (453)	10920-11372 (453)	GTG/TAG	GTG/TAG			−7	0
tRNA-Tyr (Y)	11420-11485 (66)	11389-11455 (67)			GTA	GTA	19	16
tRNA-Leu (L1)	11496-11557 (62)	11470-11536 (67)			TAG	TAG	10	14
tRNA-Ser (S2)	11558-11624 (67)	11538-11609 (72)			TGA	TGA	0	1
tRNA-Leu (L2)	11644-11708 (65)	11646-11710 (65)			TAA	TAA	19	36
tRNA-Arg (R)	11709-11775 (67)	11713-11779 (67)			TCG	TCG	0	2
*nad*5	11775-13358 (1584)	11780-13360 (1581)	GTG/TAA	ATG/TAG			−1	0
tRNA-Gly (G)	13359-13431 (73)	13365-13433 (69)			TCC	TCC	0	4
tRNA-Glu (E)	13440-13507 (68)	13451-13515 (65)			TTC	TTC	8	17
LNCR	13508-14050 (543)	13516-14014 (499)					0	0

SNCR: Short non-coding region. LNCR: Long non-coding region.

Data of *P. cervi* (PC) mt genome sequence was derived from Yan et al. (2013) [21] (GenBank accession No. KF_475773).

**Table 3:** **Comparison of nucleotides and predicted amino acids sequences between** ***Paramphistomum leydeni*** **(PL) and** ***Paramphistomum cervi*** **(PC)**

**Gene**	**nt length (bp)**		**nt diversity**	**Amino acid no.**		**Amino acid**
**/region**	**PL**	**PC**	**(%)**	**PL**	**PC**	**Diversity (%)**
*cox*3	645	645	12.25	214	214	8.88
*cyt*b	1113	1113	13.39	370	370	9.19
*nad*4L	264	264	12.88	87	87	6.90
*nad*4	1281	1281	13.66	426	426	8.69
*atp*6	516	516	11.43	171	171	10.53
*nad*2	873	873	15.23	290	290	14.14
*nad*1	897	897	11.04	298	298	7.72
*nad*3	357	357	9.80	118	118	10.17
*cox*1	1545	1545	12.30	514	514	5.25
*cox*2	582	579	9.45	193	192	9.84
*nad*6	453	453	15.89	150	150	12.67
*nad*5	1584	1581	16.10	527	526	9.49
*rrn*L	995	986	10.53	-	-	-
*rrn*S	749	749	11.67	-	-	-
LNCR	543	499	38.33	-	-	-
SNCR	64	58	35.94	-	-	-
All 22 tRNA	1446	1438	13.20	-	-	-

The gene arrangement of the mt genome of *P. leydeni* is identical to that of *P. cervi*, but is obviously different from some species of *Schistosoma*, such as *Schistosoma mansoni*, *Schistosoma spindale* and *Schistosoma haematobium* [36,39-42]. The two rumen flukes, together with *Opisthorchis* spp*.* [32,33]*, Fasciola* spp. [19,35]*, Dicrocoelium* spp*.* [20], *C. sinensis* [32,33] and *S. turkestanicum* [36]*,* share the same protein-coding gene and rRNA gene arrangement, which are interrupted by different tRNA genes or tRNA gene combinations, indicating important phylogenetic signal for Paramphistomatidae from the switched position of tRNA genes [39].

The nucleotide compositions of the whole mt genomes of two flukes reveal high T content and low C content, with T content being 44.53% in *P. leydeni* and 44.95% in *P. cervi* and C content being 9.44% in *P. leydeni* and 9.10% in *P. cervi*. The nucleotide composition of these two entire mt genomes is biased toward A and T, with an overall A + T content of 63.77% for *P. leydeni* and 63.40% for *P. cervi* respectively, which is within the range of magnitude of the trematode mt genomes (51.68% in *P. westermani* to 72.71% in *S. spindale*) [36,39-42].

The A + T content for the mt genomes of the two rumen flukes is shown in Additional file 1: Table S1. The A + T content of each gene and region range from 53.23% to 74.19% for *P. leydeni* and 52.24% to 69.84% for *P. cervi*. Both the highest and the lowest A + T content of two mt genomes exist in tRNA genes of *P. leydeni* and *P. cervi*, while the other genes and regions occupy more steady A + T content of 60.94% to 67.29% and 60.88% to 66.78%, respectively. The A + T content of 12 protein-coding genes of *P. leydeni* are generally higher than that of *P. cervi*, except for *atp*6, *nad*2, *nad*6 and *nad*5. Other than high A + T content of NCRs in Schistosomatidae (>72% in *S. spindale* and >97% in *S. haematobium*) [39], the A + T content of NCRs of Paramphistomatidae are at around 62%, with 60.94% to 63.90% in *P. leydeni*, and 62.07% to 64.33% in *P. cervi*, as shown in Additional file 1: Table S1.

### Annotation of mt genome of *P. leydeni*

In the *P. leydeni* mt genome, the open reading-frames of 12 protein-coding genes have ATG or GTG or ATA as initiation codons, TAG or TAA as termination codons. It is noticable that *P. leydeni* is the only trematode found initiating *nad*2 with ATA so far. None of the 12 genes in the mt genome of *P. cervi* uses ATA as initial codons, nor TAA as termination codons (Table 2). No incomplete terminal codons were observed in either of genomes of the two *Paramphistomum*. In the mt genomes of *P. leydeni*, 22 tRNA genes, ranging from 59 to 73 bp in size, have similar predicted secondary structures to the corresponding genes from *P. cervi* [21]. In both mt genomes, the *rrn*L gene is situated between tRNA-Thr and tRNA-Cys, and *rrn*S locates between tRNA-Cys and *cox*2 (Table 2). The length of the *rrn*L gene is 995 bp for *P. leydeni*, 9 nt longer than that in *P. cervi*. The length of the *rrn*S gene is 749 bp for both *P. leydeni* and *P. cervi*. For these two mt genomes, the long non-coding regions (LNCR) and short non-coding regions (SNCR) are situated between the tRNA-Glu and *cox*3, and *cyt*b and *nad*4L, respectively (Table 2). Though the NCRs reveal no remarkable features, it is speculated that the AT-rich domain could be connected with the replication and transcription initiation [43,44].

### Comparative analyses of mt genomes of *P. leydeni* and *P. cervi*

The magnitude of sequence difference across the entire mt genome between the two paramphistomums is 14.7% (2088 nucleotide substitutions in all), slightly larger than that between *F. hepatica* and *F. gigantica* (11.8%) [19] and *D. chinensis* and *D. dentiticum* (11.81%) [20].

For the 12 protein genes of *P. leydeni* and *P. cervi*, comparisons also reveal sequence differences at both nucleotide (13.3%, a total of 1336 nucleotide substitutions) and amino acid level (9.05%, a total of 304 amino acid substitutions), which are larger than those between *F. hepatica* and *F. gigantica* (11.6% and 9.83%, respectively) [19], and between *D. chinensis* and *D. dentriticum* (11.7% and 11.36%, respectively) [20].

A comparison of the nucleotide and amino acid sequences inferred from individual mt protein-coding genes of *P. leydeni* and *P. cervi* is shown in Table 3. The nucleotide sequence differences of 12 protein coding-genes range from 9.45% to 16.10%, with *cox*2 and *nad*5 being the most and the least conserved genes, respectively. It is notable that the *nad*5 gene is regarded as the most conserved protein-coding gene in *Dicrocoelium*, based on nucleotide sequences comparison between *D. dendriticum* and *D. chinensis* [20]. The amino acid sequence differences of *P. leydeni* and *P. cervi* range from 5.25% to 14.14%. Based on the inferred amino acid sequence differences, *cox*1 and *nad*2 are the most and the least conserved protein-coding genes respectively. It is noteworthy that the *nad*6 gene possesses the highest level of sequence difference in Fasciolidae and Dicrocoeliidae [19,20].

Nucleotide differences also exist in ribosomal RNA genes [*rrn*L (10.53%) and *rrn*S (11.67%)], tRNA genes (13.20%) and non-coding regions [LNCR (38.33%) and SNCR (35.94%)] (Table 3). Through the comparison of entire mt genomes of *P. leydeni* and *P. cervi*, *cox*2 is the most conserved gene (Table 3). It is worth noting that the most conserved gene in *Dicrocoelium* is *rrn*S [20]. Results of these comparative analyses indicate that *P. leydeni* and *P. cervi* represent distinct fluke species.

### Sliding window analysis of nucleotide variability

**Figure 2:**
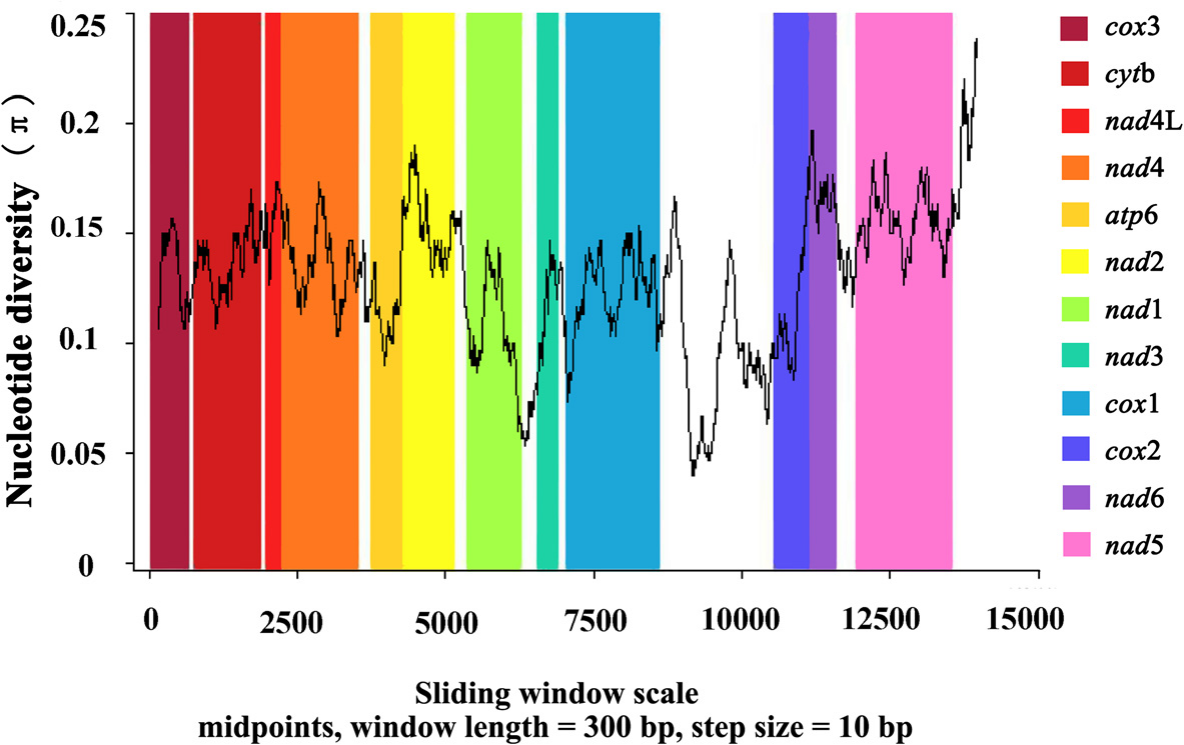
**Sliding window of nucleotide variation in complete mt genome sequences of** ***Paramphistomum leydeni*** **and** ***P. cervi*** **.** The folding line indicates nucleotide variation in a window of 300 bp (steps in 10 bp). Regions and boundaries of 12 protein-coding genes are indicated by color.

By computing the number of variable positions per unit length of gene, the sliding window indicated that the highest and lowest levels of sequence variability were within the genes *nad*5 and *cox*2, respectively. In this study, protein-coding genes of *cox*2, *nad*3 and *nad*1 are the most conserved protein-coding genes, while *nad*5, *nad*6 and *nad*2 are the least conserved (Figure 2). These results are slightly different from those among *Fasciola* spp. that *cyt*b and *nad*1 were the most conserved genes, while *nad*6, *nad*5 and *nad*4 were the least conserved [19].

### Phylogenetic analysis

**Figure 3:**
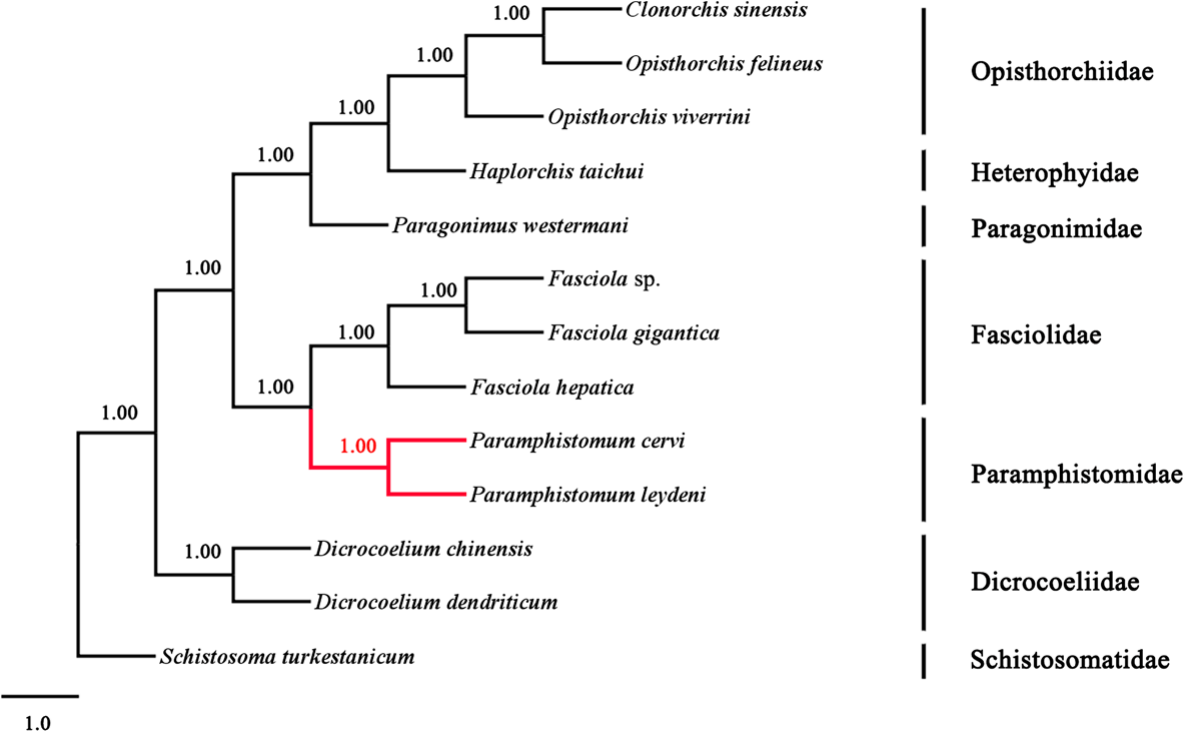
**Phylogenetic relationships of** ***Paramphistomum leydeni*** **and** ***P. cervi*** **, and other trematodes.** Phylogenetic analysis of the concatenated amino acid sequence datasets representing 12 protein-coding genes was performed by Bayesian inference (BI), using *Schistosoma turkestanicum* (HQ_283100) as an outgroup.

Phylogenetic analysis of the concatenated amino acid sequence datasets for all 12 mt proteins (Figure 3) reflected the clear genetic distinctiveness between *P. leydeni* and *P. cervi* and also the grouping of these two members of *Paramphistomum* with other members of families Opisthorchiidae, Heterophyidae, Paragonimidae, Fasciolidae, Dicrocoeliidae and Schitosomatidae, with strong nodal support (posterior probability = 1.00). The difference between the two *Paramphistomum* spp. is similar to that between *F. hepatica* and *F. gigantica* [19], *D. chinensis* and *D. dentriticum* [20], and *C. sinensis* and *O. felineus* [33] by observing the lengths of the branches. The phylogenetic analysis further confirmed that *P. leydeni* and *P. cervi* are different *Paramphistomum* species.

### Nucleotide differences in ITS-2 rDNA between *P. leydeni* and *P. cervi*

The rDNA region sequenced from individual *P. leydeni* samples was approximately 2582 bp in length, including partial 18S rDNA, complete ITS-1, complete 5.8 rDNA, complete ITS-2, and partial 28S rDNA. ITS-2 was 286 bp in length. Sequence difference in ITS-2 rDNA was 3.1% between the *P. leydeni* and *P. cervi*, which is slightly lower than that between *D. chinensis* and *D. dentriticum* (3.8-6.3%), but higher than that between *F. hepatica* and *F. gigantica* (1.7%) [19], while no sequence variation was observed within *P. leydeni* and *P. cervi*. These results provided additional strong support that *P. leydeni* and *P. cervi* are different trematode taxa.

In spite of the evidence of genetic difference between two *Paramphistomum* species, elaborate population genetic investigations still need to be conducted. Further studies could (i) explore nucleotide variation in mtDNAs among *Paramphistomum* populations in various hosts of numerous countries from different continents, (ii) establish accurate molecular tools and rapid detection methods, (iii) decipher the genomes of *Paramphistomum* using next generation sequencing (NGS) technologies. It is believed that elucidating the transcriptomes, proteomes and genomes of *Paramphistomum* would assist in future efforts in deciphering biology and taxonomy of more trematode parasites including the important family Paramphistomatidae.

## Conclusions

The present study determined the complete mt genome sequences and ITS-2 rDNA sequences of *P. leydeni*, and provided reliable genetic evidence that *P. leydeni* and *P. cervi* are closely-related but distinct paramphistome species based on mt and nuclear ribosomal DNA dataset. The accurate identification of the two rumen flukes will contribute to the diagnosis and control of paramphistomiasis. The availability of the complete mt genome sequences and nuclear rDNA sequences of *P. leydeni* could provide additional genetic markers for studies of the epidemiology, population genetics and phylogenetic systematics of trematodes.

## Additional file

Additional file 1: Table S1. Comparison of A + T content of mitochondrial genomes of *Paramphistomum leydeni* (PL) and *Paramphistomum cervi* (PC).
