# Supplementary material for: Mitochondrial and nuclear ribosomal DNA dataset supports that Paramphistomum leydeni (Trematoda: Digenea) is a distinct rumen fluke species
Source: Parasit Vectors. 2015 Apr 2;8:201. doi: 10.1186/s13071-015-0823-4 (PMC4392617; doi:10.1186/s13071-015-0823-4)
Supplement: Additional file 1: Table S1. — Comparison of A + T content of mitochondrial genomes of Paramphistomum leydeni (PL) and Paramphistomum cervi (PC). [file 13071_2015_823_MOESM1_ESM.doc]

**Additional file 1**

**Table S1 Comparison of A+T content of mitochondrial genomes of *Paramphistomum leydeni* (PL) and *Paramphistomum cervi* (PC).**

| **Gene/Region** | **A (%)** | | **T (%)** | | **G (%)** | | **C (%)** | | | **A+T (%)** | | |
| --- | --- | --- | --- | --- | --- | --- | --- | --- | --- | --- | --- | --- |
|  | **PL** | **PC** | **PL** | **PC** | **PL** | **PC** | **PL** | | **PC** | **PL** | | **PC** |
| *cox*3 | 17.83 | 15.04 | 49.46 | 50.08 | 24.81 | 27.75 | 7.91 | 7.13 | | 67.29 | 65.12 | |
| tRNA-His (H) | 26.47 | 26.09 | 38.24 | 37.68 | 29.41 | 30.43 | 5.88 | 5.80 | | 64.71 | 63.77 | |
| *cyt*b | 18.42 | 16.98 | 45.28 | 45.82 | 26.95 | 28.03 | 9.34 | 9.16 | | 63.70 | 62.80 | |
| SNCR | 17.19 | 20.69 | 43.75 | 41.38 | 29.69 | 31.03 | 9.38 | 6.90 | | 60.94 | 62.07 | |
| *nad*4L | 22.73 | 17.42 | 43.56 | 46.97 | 25.38 | 29.17 | 8.33 | 6.44 | | 66.29 | 64.39 | |
| *nad*4 | 15.85 | 15.53 | 48.56 | 48.71 | 25.37 | 26.39 | 10.23 | 9.37 | | 64.40 | 64.25 | |
| tRNA-Gln (Q) | 19.70 | 16.92 | 43.94 | 40.00 | 27.27 | 30.77 | 9.09 | 12.31 | | 63.64 | 56.92 | |
| tRNA-Phe (F) | 28.36 | 26.15 | 32.84 | 35.38 | 29.85 | 29.23 | 8.96 | 9.23 | | 61.19 | 61.54 | |
| tRNA-Met (M) | 32.31 | 32.81 | 35.38 | 35.94 | 18.46 | 17.19 | 13.85 | 14.06 | | 67.69 | 68.75 | |
| *atp*6 | 16.47 | 16.28 | 48.84 | 29.81 | 24.81 | 24.22 | 9.88 | 9.69 | | 65.31 | 66.09 | |
| *nad*2 | 14.32 | 14.89 | 51.66 | 51.89 | 26.69 | 25.66 | 7.33 | 7.56 | | 65.98 | 66.78 | |
| tRNA-Val (V) | 32.81 | 31.25 | 39.06 | 34.38 | 17.19 | 23.44 | 10.94 | 10.94 | | 71.88 | 65.62 | |
| tRNA-Ala (A) | 20.91 | 27.14 | 32.84 | 30.00 | 31.34 | 27.14 | 14.93 | 15.71 | | 53.73 | 57.14 | |
| tRNA-Asp (D) | 22.84 | 26.15 | 42.86 | 36.92 | 25.71 | 27.69 | 8.57 | 9.23 | | 65.71 | 63.08 | |
| *nad*1 | 16.16 | 15.05 | 47.71 | 48.16 | 28.54 | 29.32 | 7.58 | 7.47 | | 63.88 | 63.21 | |
| tRNA-Asn (N) | 30.30 | 27.27 | 39.39 | 40.91 | 19.70 | 22.73 | 10.61 | 9.09 | | 69.70 | 68.18 | |
| tRNA-Pro (P) | 27.27 | 20.63 | 36.36 | 39.68 | 27.27 | 30.16 | 9.09 | 9.52 | | 63.64 | 60.32 | |
| tRNA-Ile (I) | 22.58 | 22.22 | 37.10 | 38.10 | 27.42 | 26.98 | 12.90 | 12.70 | | 59.68 | 60.32 | |
| tRNA-Lys (K) | 24.24 | 25.76 | 40.91 | 40.91 | 21.21 | 21.21 | 13.64 | 12.12 | | 65.15 | 66.67 | |
| *nad*3 | 15.13 | 15.13 | 51.26 | 50.14 | 27.17 | 27.73 | 6.44 | 7.00 | | 66.39 | 65.27 | |
| tRNA-Ser (S1) | 20.34 | 20.34 | 37.29 | 37.29 | 25.42 | 27.12 | 16.95 | 15.25 | | 57.63 | 57.63 | |
| tRNA-Trp (W) | 31.25 | 31.75 | 37.50 | 38.10 | 20.31 | 19.05 | 10.94 | 11.11 | | 68.75 | 69.84 | |
| *cox*1 | 16.89 | 15.99 | 44.85 | 45.57 | 27.12 | 27.31 | 11.13 | 11.13 | | 61.75 | 61.55 | |
| tRNA-Thr (T) | 32.26 | 27.69 | 41.94 | 41.54 | 20.97 | 26.15 | 4.84 | 4.62 | | 74.19 | 69.23 | |
| *rrn*L | 27.14 | 26.04 | 37.19 | 37.59 | 25.33 | 26.75 | 10.35 | 9.63 | | 64.32 | 63.63 | |
| tRNA-Cys (C) | 29.85 | 26.67 | 37.31 | 38.33 | 20.90 | 23.33 | 11.94 | 11.67 | | 67.16 | 65.00 | |
| *rrn*S | 24.97 | 23.74 | 36.18 | 37.14 | 26.30 | 27.72 | 12.55 | 11.41 | | 61.15 | 60.88 | |
| *cox*2 | 20.10 | 19.34 | 41.75 | 41.80 | 27.49 | 28.67 | 10.65 | 10.19 | | 61.86 | 61.14 | |
| *nad*6 | 15.45 | 14.79 | 47.46 | 49.23 | 27.81 | 28.92 | 9.27 | 7.06 | | 62.91 | 64.02 | |
| tRNA-Tyr (Y) | 30.30 | 32.84 | 33.33 | 31.34 | 28.79 | 26.87 | 7.58 | 8.96 | | 63.64 | 64.18 | |
| tRNA-Leu (L1) | 22.58 | 19.40 | 30.65 | 34.33 | 30.65 | 31.34 | 16.13 | 14.93 | | 53.23 | 53.73 | |
| tRNA-Ser (S2) | 28.36 | 23.61 | 32.84 | 37.50 | 28.36 | 29.17 | 10.45 | 9.72 | | 61.19 | 61.11 | |
| tRNA-Leu (L2) | 24.62 | 24.62 | 30.77 | 30.77 | 27.69 | 27.69 | 16.92 | 16.92 | | 55.38 | 55.38 | |
| tRNA-Arg (R) | 21.21 | 20.90 | 36.36 | 31.34 | 24.24 | 32.84 | 18.18 | 14.93 | | 57.58 | 52.24 | |
| *nad*5 | 16.86 | 16.51 | 46.59 | 47.56 | 28.98 | 27.83 | 7.58 | 8.10 | | 63.45 | 64.07 | |
| tRNA-Gly (G) | 25.68 | 24.64 | 37.84 | 37.68 | 25.68 | 26.09 | 10.81 | 11.59 | | 63.51 | 62.32 | |
| tRNA-Glu (E) | 22.06 | 16.92 | 47.06 | 50.77 | 23.53 | 24.62 | 7.35 | 7.69 | | 69.12 | 67.69 | |
| LNCR | 21.92 | 25.85 | 41.99 | 38.48 | 27.99 | 27.45 | 8.10 | 8.22 | | 63.90 | 64.33 | |
